# Consumer perceptions of direct-to-consumer electronic prescription services in Queensland, Australia

**DOI:** 10.1177/20552076241294184

**Published:** 2024-11-08

**Authors:** Amelia R Cossart, Eloise Kennedy, Faith R Yong, Jodie B Hillen, Christopher R Freeman

**Affiliations:** 1 School of Pharmacy, 1288 University of Queensland, Brisbane, QLD, Australia; 2 Faculty of Medicine, 1974 The University of Queensland, Herston, QLD, Australia; 3 Metro North Hospital and Health Service, Herston, QLD, Australia

**Keywords:** Digital health, electronic prescriptions, telehealth, direct-to-consumer, health care quality, best practice care and medicines safety

## Abstract

**Background:**

Direct-to-consumer (DTC) electronic prescription services (EPS) are a novel addition to the Australian healthcare landscape. This study aimed to explore consumers’ perceptions on how this model of care supports the delivery of best-practice care.

**Method:**

Focus groups participants were recruited through social media and included adults aged 18 years or older, Queensland (Australia) residents, and interested in DTC EPS. Focus groups were conducted via Zoom^®^ and repeated until data saturation. Inductive thematic analysis was undertaken to elicit consumer perception themes from focus group discussions and field notes.

**Results:**

Three focus groups were conducted between July and August 2022 and included 13 participants of which two (15%) had previously used DTC EPS. Four major perception themes were induced: (a) Consumer responsibility. There is an assumed level of consumer health literacy leading to an unacceptable burden of responsibility on the patient; (b) System processes appear to be underdeveloped to support best-practice care; (c) Access to convenient and timely healthcare will be improved for many patients, however, out of pocket expenses may promote inequity; and (d) Service model improvements can address safety and quality concerns including integration of the model within existing national digital health platforms.

**Conclusion:**

Participants believed that DTC EPS was a valuable addition to the Australian health care landscape increasing convenient and timely access to medicines for consumers. Participants were concerned that a heavy reliance on health literacy and underdeveloped system processes may lead to unsafe prescribing.

## Introduction

In recent years, and particularly since the COVID-19 pandemic, telehealth service usage has increased in Australia.^1^ Telehealth services allow clinicians to diagnose, treat, monitor and advise consumers remotely without the need for on-site consultations.^2,3^ Telehealth leverages digital devices such as smartphones and computers for ‘virtual’ consultations.^2^ In 2020, electronic prescriptions were rolled-out nationally in Australia to support the telehealth service model of care, allowing consumers to access prescriptions from prescribers virtually.^4^ Whilst all digitally-enabled Australians can access electronic prescriptions state-based difference in Drugs and Poisons Acts dictate which types of medicines are available, and this differs between states. Since 2020, 80,000 prescribers have issued over 189 million electronic prescriptions indicating a high uptake of this service amongst prescribers and the Australian public.^5^

Australians can access electronic prescriptions via two main pathways. The first involves consultation with their regular prescriber (usually a general practitioner (GP)) either in-person or via telehealth, and the second involves accessing a direct-to-consumer electronic prescription service (DTC EPS).^3, 5^ At the time of this study DTC EPS consultations did not always involve real-time communication between the consumer and the prescriber. Using the DTC pathway, consumers complete a brief medical questionnaire and submit an online prescription request, which is reviewed at convenience by a prescriber.^3^ In the event the prescriber deems the request acceptable, an electronic prescription is issued to the consumer.^3^ In both the US and UK, models for DTC EPS and are similar to the Australian model where either a patient's current GP or an independent private on-line company can provide these service.^6–8^

To ensure best practice healthcare, medicine-related interventions introduced into the Australian healthcare landscape should align with quality principles. Australia's National Medicines Policy (NMP) offers guidance to all stakeholders, including prescribers and consumers, on best-practice care to optimise medicines use and safety.^9^ This includes providing timely and equitable access to medicines when necessary, and in accordance with a consumer's individual medical and social history. All prescribing services have their strengths and weaknesses; however, it is important to continually evaluate if these services meet the NMP to determine if and where improvements can be made.

Compared to usual care (on-site consultation with regular GP), DTC EPS have theoretical advantages for both consumer and prescriber. Consumers can access DTC EPS ‘on-demand’, from any location, thus eliminating costs and inconvenience associated with travel and waiting room time.^3^ This model of care can be particularly beneficial for consumers in rural or remote communities, where travel distance is often a barrier to seeking healthcare.^3^ Prescribers can also benefit from this form of consultation, as more flexible and less resource intensive virtual consultations, compared to in-person consultations, potentially increase the efficiency of primary care delivery.^3^

Whilst there are valid strengths to this service model compared to usual care, concerns have been raised locally and internationally about whether the quality of care (best-practice care) delivered is equivalent to usual care and acceptable to consumers.^3, 7, 8, 10^ In both the US and UK, these services have been associated low standards of care including over-prescription of antibiotics.^8, 11^

There is a lack of transparency on how DTC EPS operate in Australia, and a dearth of information on consumer and GP outcomes. There are particular concerns that DTC EPS will fragment care.^10^ The Royal Australian College of General Practitioners(RACGP) position statement for ‘on demand’ telehealth services states that to support delivery of best-practice primary care, where possible, consumers should seek prescriptions from their usual GP who is familiar with their medical and social history. This suggests that DTC EPS should be reserved for exceptional circumstances, such as when the usual GP is not available.^3^ Uncoupling prescribing from the usual care model has the potential to fragment continuity of care and increase the risk of medicine misadventure and poor health outcomes.^3, 12^

Given the Australian interest in electronic prescriptions, and the rapid uptake since 2020, there is a critical need to research consumer perceptions of the DTC EPS model of care to understand if it meets consumers’ health care expectations and best practice standards. Primary care researchers recently advocated for prioritised research into all DTC health services including the quality of services and consumer perspectives.^10^

The aim of this study was to explore consumers’ perceptions of the benefits and challenges of DTC EPS model of care in the Australian health care system.

## Methods

Virtual focus groups explored consumer perceptions of the DTC EPS model of care. A moderator facilitated focus group conversations and the COREQ framework was followed for the reporting of this study.^13^ An interpretivist approach was selected for this study as per health research traditions, adopting consumer value co-creation understandings.^14, 15^ The University of Queensland Human Research Ethics Committee approved the study protocol (2022/HE001109). The University of Queensland centrally funds licenses for Zoom^®^ and NVivo 12^©^. A third-party confidentiality agreement was held with Happy Scribe^®^.

Participants were recruited through social media (e.g. Facebook community pages and UQ on-line volunteer recruitment board) and included adults 18 years or older, residing in Queensland, and interested in using or used DTC EPS. Participants residing outside of Queensland were excluded from study participation as health system regulations and models of care can differ between Australian states. Participants provided online written consent and were compensated for their time. To preserve confidentiality of personal health information, all demographic and health service usage details were requested directly from participants prior to focus groups. To minimise bias and present a range of consumer perceptions, the demographic questionnaire did not include screening criteria.

A discussion guide was developed by one author (FY) to support consistency of moderating each focus groups (Appendix 1). FY was previously trained in qualitative study methods at the Australian Consortium for Social and Political Research (ACSPRI), including running focus groups and discussion guide development. This guide sought to generate discussion around three key areas chosen by the research team after a brief literature review by one author (EK) and team consensus on necessary areas of understanding for how consumers might perceive quality and safety for such services (see Box 1). Each focus group included four main phases: introduction, rapport building, in-depth discussion, and closure (Appendix 1). The focus group guide was piloted within the research team.

Box 1. Focus group key discussion areas.

1. Affective experiences or perceptions of DTC EPS.
2. Perceived advantages and disadvantages of the service.
3. Understanding of this service could impact continuity of care in relation to normal general practitioner (GP) medical care

Participants were shown independent third-party publicly available videos (free to air news channels) to ensure all participants were informed of the DTC EPS model of care which described the model of care, the process for requesting a prescription, as well as potential advantages and concerns with this model of care (see Appendix 1). The first video explained the service from a provider's view (Instascripts™) and the second video explored doctor's concerns with DTC prescription services. Participants were requested to individually respond to open questions about the service model using Padlet. Padlet is a free subscription app to assist collaboration using visual digital boards enabling contributors to share and display information in real-time (Padlet Product).

Due to a peak in COVID-19 cases in the community during the study period, all focus groups were run virtually via Zoom^®^. Focus group discussions were recorded and transcribed using either Zoom^®^ or Happy Scribe^®^. Field notes were taken during each focus group, and screenshots of each group's individual responses were saved, before deletion from the online platform. Focus groups were facilitated by at least two members of the research team (EK, AC and FY) with one member acting as the moderator and the other member providing support (e.g. field notes and monitoring group chat). Throughout each focus group, the moderator supported participants to present their individual opinions to the group, clarified misunderstandings and facilitated group discussions. Focus groups were run until data saturation. Saturation was decided by the research team (EK, AC, FY and CF) based on facilitator debrief sessions and iterative review of focus group data. Due to limitations in project scope, participant checking of transcripts or themes were not possible.

Focus group transcripts were de-identified and inductive thematic analysis was used to analyse results as per Braun and Clarke's methodology.^16^ Common concepts within the transcripts were initially coded using NVivo 12^©^ software, then grouped into themes and subthemes by one member of the research team (EK). To optimise comprehensive and informative theme identification, codes were further refined by the research team (EK, AC and FY) until consensus was reached. Exemplars (quotes) from the focus group transcripts were used to demonstrate each theme and subtheme.

## Results

Three virtual focus groups were run after business hours on weekdays between July and August 2022. There were at least four consumers per group, with a total of 13 participants. Each focus group ran for approximately 1 hour. The median age of participants was 32 years of age (range: 18–71 years), with 15% (*n* = 2) having previously used a DTC EPS (Table 1). Most participants resided in metropolitan areas (85%, *n* = 11) and engaged with a regular GP (85%, *n* = 11). The median number of regular medicines used by participants was 2 (range 0–10).

**Table 1.** Participant characteristics.

Characteristic	*N* = 13
Age, median (range)	32 years (18–71 years)
Gender, Female (percentage)	7 (54%)
Locality (percentage)	
Metropolitan	11 (85%)
Rural	2 (15%)
Prior use of a virtual electronic prescription service (percentage)	2 (15%)
1+ chronic health conditions (percentage)	5 (38%)
Regular medicines, median (range)	2 (0–10)
Regular general practitioner (percentage)	11 (85%)

Four major themes and 10 subthemes were elicited from the focus group discussions. The themes and subthemes are described in detail below and presented in Table 2.

**Table 2.** Themes and subthemes.

	Theme	Subtheme
1.	Consumer responsibility	Assumed high health literacy.
		Lack of active prescriber involvement.
2.	Underdeveloped system processes	Potential for misuse.
		Lack of continuity of care.
		Increased cost to consumers.
3.	Increase in healthcare accessibility	Increased accessibility.
		Time-saving for consumers and GPs.
4.	Service improvement ideas	Refining access criteria.
		Medicine-related questions should be more qualitative.
		Integration of DTC EPS into national digital health platforms.

Theme 1: Consumer Responsibility: DTC EPS place the onus of responsibility on the consumer, rather than the prescriber.

A. The DTC EPS model of care presumes a high level of consumer health literacy, reliability and accountability.

Due to the minimal input from a prescriber, in the DTC EPS model compared to usual care, consumers perceived an unacceptable burden of responsibility for consumers to make important health decisions.

Therefore, participants perceived this model of care as unlikely to be appropriate for all consumers, especially consumers with limited health literacy. As this model of care operated entirely via an online platform without verbal communication between the prescriber and the consumer, participants believed this service required a possibly unrealistic level of consumer health literacy.

*‘I think that [DTC EPS] assumes quite a high level of health literacy and understanding, and a lot of old people don’t even know what they,re on and why they’re on it. So, like, giving people this and just trusting them to answer things properly, I don’t think that's necessarily safe…’* (Focus group 1)

Participants also felt consumer-led decisions would not necessarily be reliable and accountable for alignment with best practice primary care, as consumers may not have the capacity to understand the potential health consequences of their requests. Participants raised the concern that accountability for the appropriateness of prescriptions is the responsibility of the prescriber not the consumer.

B. The perceived lack of active prescriber involvement was seen as symbolic of a checklist approach to prescribing and described as a depersonalised approach to healthcare.

Due to the perceived ‘non-existent’ interaction between the consumer and prescriber, participants asserted that a therapeutic relationship could not be fostered.

*‘I think they [the doctors] should take a bit more of an involved approach, but it's hard to do that when you don’t even have, like, a phone conversation, or like a virtual conversation’.* (Focus group 3)

Participants felt that consumers may not receive the same care as the care provided in a face-to-face or telehealth consultation. The lack of prescriber–consumer real-time communication in the DTC EPS model of care led participants to perceive this service to be more ‘transactional’ and less consumer centred, as the prescriber was unlikely to have access to full medical and social records. Participants perceived that it would be challenging to meet best practice primary care using the DTC EPS as prescribers will not have access to information to inform appropriate prescribing aligned with consumer's health needs, experiences, and lifestyle.

‘*It shouldn’t just be a tick and flick. It seems to me it's kind of a bit of a tick and flick, which is, you know, when it's virtual and you don’t know the person, you don’t know that history*’. (Focus group 3)

Theme 2: Underdeveloped system processes: System processes appear to be underdeveloped to foster individualised care, medicine safety and equity.

A. The model of care could potentially be misused by consumers and prescribers, who may take advantage of the perceived checklist approach to prescribing algorithms exposed by this model.

A major discussion point for participants was the increased likelihood for consumers and healthcare professionals to misuse DTC EPS.

‘*I think there are dodgy people, but there are also dodgy, like, doctors… You could also have a dodgy pharmacist. So, it's like you could just, kind of, be taking an extra safety barrier out by removing an in-person component*’. (Focus group 1)

Participants perceived that this model of care compromised the prescriber–consumer relationship, implying that in usual care, prescribers would normally pick up on truant behaviours. As this model of care minimises interpersonal interaction, including verbal and nonverbal communication, it does not accommodate the implicit expectation that prescribers would use cues to ensure appropriate prescribing.

‘*people might eventually figure out how to abuse the system, give the answers they need to give to get what they want…*’ (Focus group 1)

Participants also felt that a lack of transparency during a consultation meant that EPS could potentially attract consumers looking to use a prescription service for illegal or therapeutically inappropriate (self-seeking) reasons. They implied the simple checklist approach represented in the model of care could be equated to specific steps in prescribing algorithms, which could then be learned and misused in a detrimental way.

‘*I don’t trust that people wouldn’t abuse it. I think that opportunists might find a way to abuse it…*’ (Focus group 3)

B. The DTC EPS model of care represented a breakdown in the continuity of care when presented as an isolated service model, compared to the rest of the healthcare system.

Since the communication was a single point in time, participants believed that the prescriber in the DTC EPS model of care could not support continuity of care for the consumer. Of particular concern was the lack of facilitated follow-up with a consumer's usual prescriber. Participants stated it was common for medicines to cause side effects; and typically, when prescribed a new medicine, the usual model of care (a traditional consultation) encourages a follow-up appointment to be scheduled to understand the success or lack thereof for the prescribed treatment. The probable lack of interconnectivity with regular GP records or shared health records worried participants, who felt their normal prescriber should be communicated with or involved in some way.

*‘Without that same follow-up from a GP, for example, and having that communication as to what side effects they’re having or that it may not work right away so it's a bit of, like, a breakup in the continuity of care for the patient – like, there's no line [i.e. process] for someone to go down if they use this service’.* (Focus group *3*)

C. This service could increase the cost to consumers if it was a privatised consultation not paid for by Medicare, which may reduce equity.

Participants felt the cost of this service may impede access for some consumers. At the time of this study, this model of care was not subsidised by the government and thus consumers were ineligible for a rebate. Participants felt that service costs could negate the perceived time savings and its accessibility advantages. Participants believed that the out-of-pocket costs associated with these services may be a barrier to access for Australians reliant on subsidised healthcare.

*‘Can they subsidise it through Medicare and things like that? Because people can only get scripts through going to a GP and they’re getting a Medicare rebate because they can't afford it otherwise… is it really accessible if it can't be cost covered’?* (Focus group 1)

Theme 3: Increase in access to healthcare and improved GP workload:

A. The DTC EPS model of care may simplify access to healthcare for some consumers by increasing convenience and ‘saving time’.

Participants commented that overall, DTC EPS would be beneficial to the majority of consumers who are time-poor, as they increase medicine accessibility and convenience. Scheduling a traditional appointment was perceived to be difficult: consumers were described as time-poor by participants, and this worsened with the increasing demands on the healthcare system and a lack of available last-minute appointments.

*‘… if it's something that you take really regularly, and you know that you don’t have any side effects to it, or you are happy with that medicine, then I think it's a great way to access your repeat prescription because it just makes it an easier way to get a hold of them… especially if you can’t get in [to see your GP] and you have to see somebody different’.* (Focus group *2*)

DTC EPS were perceived to be a convenient and beneficial addition to the Australian healthcare landscape, as an alternative to GP visits for repeat prescriptions (which were implied to be mostly unnecessary and inconvenient for both consumers and prescribers).

Compared to a usual consultation, participants felt that they would waste less time, which was seen as a major benefit of the service model.

*‘I think that it's a good idea in the fact that it definitely saves time, and I think everyone is time poor’.* (Focus group *3*)

B. The DTC EPS model of care has potential to improve GP workload.

Further, this model of care was thought to improve GP workload by reducing the number of appointments for prescription-based requests, and it was assumed that GPs could then better prioritise their time for other clinical issues or activities. Attending a traditional consultation for a prescription was then raised by some participants to be an unnecessary burden on both GPs and consumers.

*‘People who just need a repeat without any other consultation needed, it just seems like a waste of time to me. I’ve often thought that myself. A waste of my time and the GP's time. So, I think it's a win-win from that point of view’.* (Focus group 3)

Theme 4: Suggested areas for improvement of the safety, accountability, and integration of the DTC EPS model of care within the Australian healthcare system.

A. Refine eligibility criteria to improve safety.

Participants felt the consumer eligibility criteria for the service model should be stricter to avoid potential misuse and improve consumer safety. Participants discussed how refining the eligibility for service usage may enable more appropriate use by specific consumer populations (e.g. those on stable medicines to treat chronic diseases), whilst dissuading potential for misuse by consumers and healthcare providers. The participants defined misuse as persons taking advantage of the service, requesting a prescription medicine for a purpose or in a manner other than intended.

*‘You might want to tighten the parameters around what people can access, and maybe it should only be for repeat prescriptions… if you kept it limited to repeat prescriptions that would really limit the opportunity to abuse it*’. (Focus group 3)

Participants explained that the overall safety of the DTC EPS model of care could be improved using the following measures: the service should only provide repeat prescriptions; identity verification methods (such as the supply of primary identity documents) should be used prior to prescription requests being sent for prescriber assessment, and the model of care should be limited to one account per verified individual.

B. Improve medicine-related questions to align with best-practice care.

Participants felt that the number and type of medicine-related questions should be increased to enhance the depth/scope of the consultation. Participants were concerned that it would be difficult for a prescriber working virtually and asynchronously to determine the appropriateness of a consumer's request using the information recorded in the current brief on-line questionnaire. This measure was perceived to be qualitatively different to a medical history they expected a GP to assess, document, or have access to during a traditional consultation. Without the richness of an interpersonal interaction and access to a current medical history, participants felt that there needed to be improved approaches to communication to support consumer safety and align with best practice care. The use of only closed questions, for example, appeared to be insufficient.

*‘I think the question should be open and depending on different kinds of medicine for them [the patients]. Yeah, I think this should be kind of in a conversation*’. (Focus group 2)

Participants believed that the sole use of closed questions increased the risk of misdiagnosis and compromised consumer safety.

*‘If there's only a very short questionnaire… if it's just yes or no, and there's not like that ability for the GP, even though it's being checked by a GP, to follow up questions to get more information, there's definitely an opportunity for misdiagnosis and giving medicines that perhaps are needed to be given a face-to-face consultation’.* (Focus group *3*)

C. Integrate DTC EPS with the current digital health platforms.

Participants felt that the siloed nature of DTC EPS risked continuity of care and suggested the integration of this service with current digital health platforms, such as My Health Record, could facilitate best practice care. By reviewing a consumer's health records, prescribers could make more informed decisions for the appropriateness and safety of medicines for each consumer at the time of prescribing.

*‘Medical reports (and prescriptions) should be linked into the health care system to it can be reviewed at any time’.* (Focus Group 1)

## Discussion

To our knowledge, this was the first Australian study to investigate consumer perceptions of DTC EPS and provide unique insights into whether these services meet consumer health care expectations and best practice quality care. This study found that whilst consumers perceived these services to potentially address several gaps in health care delivery, there were concerns raised regarding the overall safety and quality of these services in delivering best practice care.

In this study, participants were concerned that the service may not be appropriate for consumers with low health literacy. Participants felt that in the case of low health literacy, consumers would be unable to effectively understand and communicate their health care needs leading to potentially inappropriate requests. In the case of DTC EPS, the prescriber is unable to validate health care needs at the time of issuing a prescription which places the responsibility on the consumer to be well informed regarding their health care needs. These perceptions are supported by previous research which shows that the majority of Australians have low health literacy which can lead to poor health decisions.^12, 17, 18^ Studies in Australia exploring consumer health literacy have found that less than one quarter of Australians (23%) strongly agreed that they had sufficient information to manage their health.^17, 18^ In addition, prescribers are aware that consumers often lack sufficient understanding of their health view with best practice primary care standards prioritising knowledge dissemination to ensure the consumer is effectively informed to manage their medicines.^3, 9, 12^ At the time of this study, DTC EPS did not support comprehensive communication between the consumer and prescriber, and together with our national low level of health literacy, this suggests there could be gaps in information collected, or misinformation from consumers, increasing the risk of inappropriate prescribing and potential medicine-related harms. Since September 2023, the Medical Board of Australia guidelines state that DTC EPS must include a synchronous consultation. Given the lack of historical data and the infancy of this recommendation, it is premature to report if this change has improved the quality of the service.^10^

Participants were also concerned that due to the immaturity of the current system, there is potential for a lack of end-user accountability. To increase accessibility to medicines, there are currently few restrictions on the types of medicines available and how often they can be prescribed.^5^ In addition, the checklist questionnaire is brief, meaning the criteria for assessing the suitability of a prescription request is minimal. Participants felt, in its current form, the system is open to ‘gaming’ from both consumers and prescribers, which may lead to requesting and or prescribing medicines in circumstances that are not clinically valid, unethical or unlawful.

It was also perceived that due to the siloed nature of the service, prescriber's ability to provide continuity of care is diminished. In recent years, there has been the roll-out of several state and national health platforms to allow healthcare practitioners to share and access more comprehensive consumer health data. DTC EPS services are privately run services (i.e. not government subsidised) and therefore, they do not have access nor contribute to these health data, and instead rely on accurate and honest consumer recall.^19, 20^ Ideally both consumer-reported and healthcare data would be used to triangulate and verify health information. Integration of DTC EPS with national healthcare digital platforms, such as My Health Record, would be a logical step for improving continuity of care across all healthcare settings.

Participants were concerned with the out-of-pocket costs to consumers to use this service as it is not currently subsidised like on-site or telehealth consultations. This would be particularly impactful for low-income consumers who rely on highly subsidised GP services (e.g. bulk billing with nil out of pocket cost). Market forces will dictate consumer choice in this space, however, higher out of pocket expenses will make this service inaccessible to consumers with limited financial resources, creating potential inequity in health care provision.

Participants saw the potential of DTC EPS in closing important gaps in healthcare delivery in Australia. This included the benefits of convenience and timely access for busy consumers and potentially removing the stress associated with replete or expired prescriptions. Further, this service model may reduce the workload for overburdened GPs, potentially freeing up GP time to reinvest in clinical care.

At the time of this study, DTC EPS consultations relied solely on questionnaires without verbal communication to support the consultation. As highlighted by participants, without verbal communication, the likelihood of misunderstandings or misinterpretation increases, and research shows this risk is particularly heightened for culturally diverse, health illiterate, and disadvantaged populations.^19, 21, 22^ Whilst it was assumed every effort would be made to communicate all relevant health-related information prior to the prescriber's assessment, it was suggested by participants that the lack of verbal communication may negatively affect the overall quality of care provided. Further, without built-in opportunities for prescribers to establish rapport, clarify facts or gather relevant health information, this model of care relies on consumers to accurately and honestly recall and disclose pertinent medical information to ensure appropriate prescribing.^19, 20^ As mentioned previously, the recent addition of synchronous consultations to the service model may address these concerns.

International research has explored patient preferences for DTC telehealth services.^23^ A nationwide study of over 4000 US consumers reported that over half would prefer to have an established relationship with a practitioner rather than use DTC consultations and nearly two-thirds preferred practitioners have full access to their medical records at the time of consultation.^23^ Another US study explored the quality of antibiotic prescribing between DTC (Teladoc) users and non-users and found that DTC performed poorly with respect to appropriateness. These findings align with the preferences and perceptions elicited from the focus group discussions in our study.

With respect to aligning with Australia's NMP, participants felt that this service model increased accessibility to medicines. However, safety of this service model could be improved through stricter eligibility (e.g. patients on stable medicines to treat chronic disease) and requirements for patient identification to ensure medicines are only prescribed for intended individuals. To improve quality of care, the on-line questionnaire should capture all relevant clinical information to support good prescribing practices and integration of the DTC EPS with My Health Record could improve continuity of care across health care settings.

The main limitation of this study was the lack of diversity of our participants restricting the generalisability of our findings to the Australian population. Only two of the participants had previously used the service and therefore the results cannot be interpreted as a reflection of user experience. In addition, our participants were recruited based on their interest in the topic which may indicate a higher digital literacy than the general population leading to an under-representation of challenges with using the DTC EPS. Our findings may not reflect consumer's experiences in other states or rural and remote locations as all participants resided in Queensland and the majority lived in metropolitan Brisbane. The focus group guide was not externally validated for reliability or content, but used best-practice methodology for planning and running focus groups.^24^

Replicating this study to include a more diverse and inclusive representation of the general public's perception of and experiences with this model of care, specifically including more people who have used the service, would be beneficial. As the utilisation of DTC EPS grows, more Australians will be exposed to the inherent benefits and risks. Ongoing monitoring of the alignment between consumer expectations and experiences together with associated health outcomes will inform the contribution of this service to meeting the evolving needs of consumers and prescribers.

## Conclusion

This study identified that consumers have both positive and negative perceptions of DTC EPS. Participants perceived the service to be time-saving and convenient thereby increasing access to medicines. However, participants were concerned by the unrealistic expectations of health literacy, fragmented care, and end-user costs. To improve the quality of this service, participants recommended more stringent eligibility and access criteria, as well as integration of the service model into national health data platforms.

## Supplemental Material

sj-pdf-1-dhj-10.1177_20552076241294184 - Supplemental material for Consumer perceptions of direct-to-consumer electronic prescription services in Queensland, Australia Supplemental material, sj-pdf-1-dhj-10.1177_20552076241294184 for Consumer perceptions of direct-to-consumer electronic prescription services in Queensland, Australia by Amelia R Cossart, Eloise Kennedy, Faith R Yong, Jodie B Hillen and Christopher R Freeman in DIGITAL HEALTH

## Appendix 1: Focus group guide

### Structure of focus group

The focus group will include four primary stages as follows:

**Table:**

Stage of focus group	Role of facilitator	Time
Introduction	Facilitator will give an overview of the goals and purpose of the discussion. Participants will be asked to introduce themselves and write their name on a sticky name badge.	10 minutes
Rapport building stage	Facilitator will host an activity that allows participants to provide individual answers. This provides a starting point for the discussion.	20 minutes
In-depth discussion	Facilitator will begin by asking questions related to the main purpose of the focus group, encouraging conversation that reveals participants’ thoughts and feelings. This is where the key data related to the study aim will be collected.	20 minutes
Closure	Facilitator will summarise the impressions or conclusions from the discussion and participants will clarify and confirm the information. The facilitator will answer any remaining questions from participants. Facilitator will thank the participants and indicate next steps (if applicable).	10 minutes

*The following domains have been identified as topics of interest to be raised during the discussion group. For each domain, a ‘trigger’ activity and 2–4 open-ended questions were formulated:*

- *Overall emotional affect*
- *Access to medical care*
- *Patient safety*

### Preamble

Thank you for agreeing to participate in this focus group discussion. We really appreciate your willingness to participate. We want to find out your experiences and opinions about the safety and accessibility of these new, electronic prescription services. The information provided will help us understand how these services are experienced by consumers. You are in a group with other consumers who have similarly used this experience, but each of you come with your own views and ideas. We need your input and want you to share your honest and open thoughts with us.

### Focus group rules

1. WE WANT YOU TO DO THE TALKING
  - We would like everyone to participate and share their thoughts. I may call on you if I haven’t heard from you in a whilst.
2. THERE ARE NO RIGHT OR WRONG ANSWERS
  - All persons experience and opinion is important.
  - Speak up whether you agree or disagree.
  - We want to hear a wide range of opinions.
  - There is no right or wrong answer.
3. WHAT IS SAID IN THIS ROOM STAYS HERE
  - We want you to feel comfortable sharing when sensitive issues come up.
4. WE WILL BE RECORDING THE GROUP
  - We want to capture everything you have to say.
  - The session will be recorded but your responses to the questions will be anonymised. Nothing you say will be able to be directly linked back to you.

### Focus group question guide

#### Introduction activity

Can we please have all video cameras on, if possible? I understand that internet bandwidth doesn’t always allow this, so if you can’t, please at minimum use the zoom function that allows you to put your hand up or give reactions, so we know you’d like to speak. Ok, let's give it a try: Hands up how many of us here have used an online prescription service before! How many haven’t?

Great! Before we properly get into the discussion, can you please each introduce yourself, tell us where you’re joining from and a random fact about the place/town you’re living.

#### Rapport building stage

1. We’re going to watch this video that explains the service we’re discussing today: https://www.youtube.com/watch?v=RdBoTl__jvs&t=37s *NB: if this triggers discussion, let them talk*.
2. I’m going to send you a link on the chat that will allow you to post your answers. My question is, how would you feel about using the online prescription service?
  - I’ll give you a minute to write your answer on Padlet.
  - I’ll get you each to present what you wrote for each section.
  - Great! I’m going to reorganise these sticky notes according to what I can see is positive, neutral and negative towards the service. If you disagree, please move the sticky note to where you think it should go.
  - Any comments about what others have said? Feel free to upvote other people's responses if you agree.

#### In-depth discussion

3. Next, on this NEW Padlet, can you write down what you consider would be the advantages and disadvantages of the virtual electronic prescription services? One point per sticky note, please!
  - Please present your notes to the group.
  - I’m going to group these into similar categories. Do you agree?
  - I’m opening it up to the group to comment on these advantages and disadvantages.
4. Now, I’m going to play you a short video. This video from ABC News explains some of the concerns doctors have regarding these online prescription services we’re discussing today. After watching the video, we want to get your thoughts.
  - https://www.youtube.com/watch?v=-BKjqxMmyKY After seeing this video, what are your immediate thoughts? (to stimulate discussion, a clarification question may be: do you think about continuity of care when thinking about online prescription services?)
  - How do you feel about doctor responsibilities with these online prescription services?
  - What do you think is the patient's responsibility when using these online prescription services, if anything?
  - What do you think could help with the safety of online prescription services?

*(If there is time, do this as a Padlet activity too, with the categorisation and summarisation of their thoughts at the end.)*

#### Closure

Thank you for contributing to this study. We will be transcribing the audio recording and deidentifying the transcripts. If you opted in to receiving the results of this study, we will be in touch with you to provide you with a report. The results will be written up as an end of year student report, may be published in academic and/or media publications, and possibly presented at student or academic conferences.
